# A Path Planning Method for Intelligent Ships Based on the Improved Artificial Potential Field Algorithm

**DOI:** 10.3390/s26144569

**Published:** 2026-07-19

**Authors:** Xiao Liu, Hua Deng, Xingya Zhao, Kexin Xu, Deqing Yu

**Affiliations:** 1 Navigation College, Jiangsu Maritime Institute, Nanjing 211100, China; lxiao@whut.edu.cn (X.L.); denghua5852@163.com (H.D.); 2 School of Navigation, Wuhan University of Technology, Wuhan 430063, China; zhaoxingya@whut.edu.cn; 3 College of Energy Engineering, Zhejiang University, Hangzhou 310027, China

**Keywords:** improved artificial potential field, velocity obstacle, ship domain, collision risk index, COLREGs

## Abstract

Path planning for unmanned ships has become an important research topic in recent years. To enhance navigation safety and reduce collision risk, this study proposes an improved artificial potential field (IAPF) method. A route gravitational force is introduced to guide the ship back to the planned route after collision avoidance, while the repulsive force is optimized to improve path smoothness and obstacle-avoidance stability. A collision-risk-index-based repulsive force is further developed for dynamic obstacle avoidance, and its direction is modified according to the COLREGs. In addition, a time-sequential rolling path-planning framework integrating the APF and velocity obstacle algorithms is proposed to suppress path oscillation and adapt to target-ship maneuvers. The method is validated in head-on, crossing, and multiple-obstacle scenarios. The minimum passing distances are 1064.07 m, 1072.98 m, and 1109.66 m, respectively, and the maximum decision time is 148 ms. The results demonstrate that the proposed method can generate smooth, COLREGs-compliant paths, avoid local minima, adapt to dynamic encounters, and satisfy real-time collision-avoidance requirements.

## 1. Introduction

As cutting-edge technologies like artificial intelligence continue to advance rapidly, the maritime industry is undergoing a comprehensive transformation towards intelligence [1,2]. Intelligent ships not only enhance navigation safety but also reduce operational costs and protect the marine environment [3]. As a critical technology for intelligent ships, path planning aims to design routes to avoid collisions, which should comply with the International Regulations for Preventing Collisions at Sea (COLREGs).

Traditional path planning algorithms include the A* algorithm [4], Dijkstra algorithm [5], ant colony optimization (ACO) algorithm [6], particle swarm optimization algorithm [7], artificial potential field (APF) algorithm [8] and so on. The APF algorithm is widely used due to its low computational complexity and excellent real-time performance among those methods. Considering the shortcomings of traditional APF, such as local minimum problems, many researchers have conducted extensive research on algorithm improvement to achieve more effective results.

### 1.1. Related Works

#### 1.1.1. Local Minimum Problem

The local minimum problem occurs when the gravitational force and repulsive force balance out, preventing the ship from moving toward its target and effectively avoiding obstacles, thereby compromising the safety and efficiency of navigation. Common methods for escaping local minima include the use of perturbation strategies, the introduction of global planning algorithms, and modifications to potential field functions.

Xiao et al. proposed a random perturbation potential energy perturbation mechanism to break the symmetry of local potential fields and help ships escape the local minimum trap [9]. Ning et al. proposed the simulated annealing algorithm to solve the local minimum problem [10]. However, perturbation strategies typically involve introducing random perturbations to escape local minima, but these perturbations do not fully consider collision avoidance rules. In addition, perturbation strategies may cause the path to jump near local minima, requiring subsequent fitting to smooth the path. This not only increases computational complexity but also affects the real-time performance of the algorithm, making it difficult to ensure an efficient collision avoidance response.

Li et al. proposed an optimized APF-ACO algorithm based on the roulette wheel selection method to select the next node to be accessed to solve the local optimality problem [11]. Sang et al., proposed a novel deterministic algorithm based on the APF and A* algorithm [12], which helps ships overcome the local minima by switching target points. However, the introduction of global planning algorithms usually requires consideration of the state and objectives of the entire environment, with a large computational load, especially when the environment is complex or there are many obstacles, which may significantly increase the computation time and affect real-time performance. Moreover, global planning algorithms are typically based on static environmental assumptions, making it difficult to quickly respond to dynamic changes in targets or obstacles.

Puriyanto et al. addressed the local optimization problem by introducing auxiliary functions [13], which modify the resultant force values generated by forces acting along each axis. By improving the potential field function to solve the minimum problem, real-time performance can be ensured without significantly increasing computational complexity. However, the improvement method of the potential field function has a direct impact on the collision avoidance effect, so the accuracy and strategy requirements for the improvement are high.

#### 1.1.2. Dynamic Obstacle Handling

In traditional APF algorithms, the direction of repulsive force is determined only by the relative position between the ship and the obstacle. However, for dynamic obstacles, the requirements of collision avoidance rules also need to be considered. In recent years, many APF methods that consider COLREGs have been proposed [14,15,16,17].

However, some studies only focus on the compliance of the avoidance direction in different encounter scenarios, neglecting the timing of collision avoidance [11]. The COLREGs require early avoidance measures to reduce collision risks, but in these studies, the timing of ship avoidance is often late, failing to avoid promptly when collision risks first appear, which may lead to weakened collision avoidance effects and even collisions.

In addition, some studies assume that the target ship maintains a fixed heading and speed [18,19,20]. However, when the motion state of the target ship changes, these assumptions may no longer hold, leading to the failure of relevant algorithms and the inability to effectively meet the collision avoidance requirements of dynamic changes.

#### 1.1.3. Path Smoothing

The path generated by the traditional artificial potential field method is usually more linear and does not conform to ship dynamics. To satisfy the dynamic requirements of ship maneuvering, Chen et al. studied the mechanism in the fusion of the APF algorithm and ship maneuvering motion model [21], and proposed a path planning method considering ship maneuverability. Chen et al. realized an optimal collision avoidance path satisfying ship dynamics [22], by combining the optimal field prediction strategy and the Nomoto model.

Although some of the paths obtained from research conform to the principles of ship dynamics, there are instances of repetition. This path design may not only lead to unnecessary fuel consumption and time waste but also affect navigation efficiency and pose potential risks to navigation safety.

#### 1.1.4. Research in Other Areas

Some scholars have developed hybrid algorithms that combine the APF algorithm with other techniques to harness the strengths of various approaches while mitigating their weaknesses. Zhu et al. proposed an integrated path planning algorithm based on velocity synthesis and APF algorithms, which shortened the path’s length and improved the efficiency [23]. Yang et al. (2024) proposed an enhanced A* algorithm incorporating APF for USV path planning, which excludes redundant hazardous nodes to reduce computational complexity [24]. Song et al. (2018) proposed a dynamic collision avoidance algorithm based on APF, VO, and particle swarm optimisation algorithms [25]. Wang et al. improved autonomous collision avoidance path planning by integrating the APF algorithm to refine the action space and reward function within the improved DDPG algorithm [26,27]. Fu et al. proposed an APF-ACO algorithm that shows enhanced convergence speed and more effective path planning outcomes [28]. However, these hybrid algorithms increase computational complexity and cannot guarantee real-time path planning in complex environments. To ensure a ship can safely navigate within the confines of a restricted waterway, Wang and Im proposed a deceleration strategy for quick speed adjustments and introduced a boundary-repulsive field based on channel boundaries [29]. Guo et al. proposed an improved autonomous collision avoidance algorithm based on the APF algorithm [30], which establishes five potential field functions to calculate the combined force.

### 1.2. Major Contribution

Although the improved artificial potential field method has made some progress in handling local minima, dynamic obstacle avoidance, and path smoothing constraints, there are still some limitations. To address these issues, this paper aims to propose the following improvements:

(1) Local minima problem: The COLREGs are considered to adjust the direction of repulsion to point towards the port or starboard side of the ship. At the same time, the “most dangerous obstacle” strategy is proposed, which means that the current decision of the ship is only affected by the most dangerous obstacle, thereby optimizing collision avoidance behavior.
(2) Dynamic obstacle handling: To effectively solve the problem of too-late collision avoidance, the collision risk function is introduced to improve the obstacle repulsion function, and the risk repulsion function is introduced. A temporal rolling decision framework is proposed for the random motion problem of the target ship, which can adapt to the random motion characteristics of the target ship.
(3) Path smoothing: The response-type nonlinear ship motion model is to make the planned path satisfy ship dynamics. A time-rolling path planning framework aligned with maritime practices is proposed by integrating the APF and VO algorithms.

The rest of this paper is organized as follows. Section 2 provides an overview of the improved path planning framework based on the APF algorithm. Section 3 details the enhancements made to the APF algorithm. Section 4 presents and analyzes the simulation results. Section 5 offers the conclusions drawn from the study.

## 2. Framework of the Research

**Figure 1:**
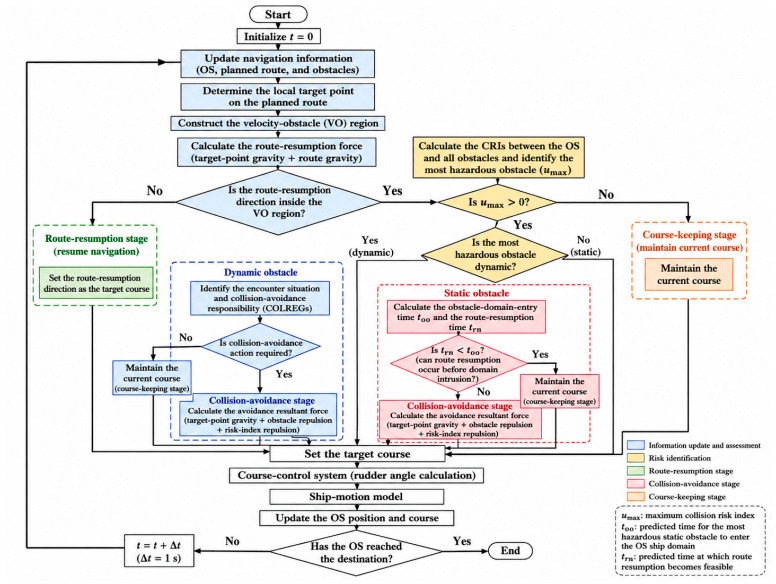
Flowchart of the proposed time-sequential rolling path-planning framework.

In maritime practice, a planned route is generally established before departure and is designed to avoid known static obstacles. Therefore, this study focuses on local path planning when the own ship (OS) encounters dynamic ships or unexpected static obstacles during navigation. The proposed framework operates in a time-sequential rolling manner with an update interval of 1 s. At each decision cycle, the navigation information is updated and the target course is recalculated according to the current obstacle distribution and collision risk. Consequently, the OS can promptly respond to changes in the motion states of surrounding target ships. The overall decision-making process is shown in Figure 1 and is described as follows.

Step 1: Update navigation information and assess the feasibility of route resumption.

At the beginning of each decision cycle, the information on the OS, planned route, target ships, and static obstacles is updated. The current local target point on the planned route is then determined, and the velocity-obstacle region is constructed using the VO algorithm. The route-resumption resultant force, composed of the target-point gravitational force and the route gravitational force, is calculated. If the direction of this resultant force lies outside the velocity-obstacle region, it is regarded as navigable and is selected as the target course. The OS then enters the route-resumption stage. Otherwise, collision-risk assessment is performed.

Step 2: Calculate the collision risk and identify the most hazardous obstacle.

The collision risk indices between the OS and all surrounding obstacles are calculated, and the obstacle with the maximum collision risk index, $u_{max}$, is identified as the most hazardous obstacle. If $u_{max}$ = 0, no immediate collision-avoidance action is required, and the OS maintains its current course. If $u_{max}$ > 0, the type of the most hazardous obstacle is identified.

Step 3: Determine the navigation action for a dynamic obstacle.

If the most hazardous obstacle is dynamic, the encounter situation and the collision-avoidance responsibility of the OS are determined in accordance with the COLREGs. If collision-avoidance action is required, the avoidance resultant force is calculated by combining the target-point gravitational force, the obstacle repulsive force, and the risk-index-based repulsive force. The direction of this resultant force is then selected as the target course. If collision-avoidance action is not required, the OS maintains its current course.

Step 4: Determine the navigation action for a static obstacle.

If the most hazardous obstacle is static, the predicted time for the obstacle to enter the OS domain, $t_{oo}$, and the predicted time at which route resumption becomes feasible, $t_{rn}$, are calculated. If $t_{rn}<t_{oo}$, the OS can safely maintain its current course and wait for the opportunity to resume navigation. Otherwise, the static obstacle may enter the OS ship domain before route resumption becomes feasible. In this case, the avoidance resultant force is calculated, and its direction is selected as the target course.

Step 5: Execute course control and update the OS motion state.

After the target course is determined, it is transmitted to the course-control system to calculate the required rudder angle. The rudder command is then applied to the ship-motion model to update the position and course of the OS.

Step 6: Check the termination condition.

If the OS has reached the destination of the planned route, the path-planning process terminates. Otherwise, the simulation time is updated according to t = t+Δt, Δt = 1s, and the procedure returns to Step 1 for the next decision cycle.

The proposed path-planning process includes three navigation stages:

(1) Collision-avoidance stage.

A collision risk exists and the OS is required to take collision-avoidance action. The target course is determined by the avoidance resultant force composed of the target-point gravitational force, the obstacle repulsive force, and the risk-index-based repulsive force.

(2) Course-keeping stage.

The OS does not require immediate collision-avoidance action, while the condition for route resumption has not yet been satisfied. Therefore, the current course is retained as the target course while the OS waits for a safe opportunity to resume navigation.

(3) Route-resumption stage.

The direction of the resultant force composed of the target-point gravitational force and the route gravitational force lies outside the velocity-obstacle region. This direction is selected as the target course to guide the OS back toward the planned route.

## 3. Methodology

### 3.1. Correlation Theory

#### 3.1.1. Ship Domain

**Figure 2:**
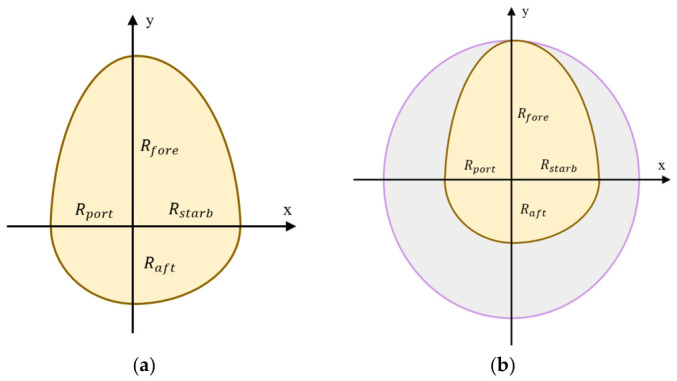
(**a**) Quaternion ship domain; (**b**) Ship domain in this study.

The ship domain refers to the safe space around a ship that must be maintained to prevent collisions with other obstacles. A quaternion ship domain is used in this study which considers factors such as ship maneuvering performance, ship size, COLREGs, and speed, as shown in Figure 2a.

Where $R_{fore}$ and $R_{aft}$ represent the radius lengths in the forward and backward directions, respectively. $R_{starb}$ and $R_{port}$ represent the radius lengths in the starboard and port directions, respectively. The calculation method is determined as Equation (1).

(1) $$\left\{\begin{array}{c} R_{fore}=\left(1+1.34\sqrt{k_{AD}^{2}+{\left(0.5k_{DT}\right)}^{2}}\right)L\\ R_{aft}=(1+0.67\sqrt{k_{AD}^{2}+{\left(0.5k_{DT}\right)}^{2}})L\\ R_{starb}=(0.2+k_{AD})L\\ R_{port}=(0.2+0.75k_{AD})L \end{array}\right.$$

where L represents the length of the ship. $k_{AD}$ represents the advance. $k_{DT}$ represents the tactical diameter, which can be calculated using empirical formulas, as in Equation (2).

(2) $$\left\{\begin{array}{c} k_{AD}=10^{\left(0.3591\log_{10}\left(v\right)+0.0952\right)}\\ k_{DT}=10^{\left(0.5441\log_{10}\left(v\right)-0.0795\right)} \end{array}\right.$$

where v represents the speed of the ship, $\log_{10}\left(\cdot \right)$ denotes the base-10 logarithm.

For the convenience of calculation, a circle with the radius of $R_{fore}$ is used as the ship domain, as shown in Figure 2b.

#### 3.1.2. Collision Risk Index Model

The collision risk index (CRI) is determined by factors such as dynamic situation and ship maneuverability, indicating the collision risk level between the two ships and the urgency of taking avoidance measures. It can be divided into temporal collision risk index (TCRI) and spatial collision risk index (SCRI), as in Equation (3). SCRI refers to the scenario where if both the OS and the target ship maintain their current speed and course, the target ship will eventually invade the OS’s domain, as in Equation (4). TCRI quantifies the urgency of collision avoidance maneuvers by estimating the time remaining until the target ship enters the OS’s ship domain, as in Equation (5).

(3) $$u=u_{s}u_{t}$$

(4) $$u_{s}=\left\{\begin{array}{c} 1\;\exists t<1800s,(x,y)\in D_{os}\\ 0\;\forall t<1800s,(x,y)\notin D_{os} \end{array}\right.$$

(5) $$u_{t}=\left\{\begin{array}{cc} 1 & \;T_{is}\leq 0\\ {\left(1-\frac{T_{is}}{T_{t}}\right)}^{1.68} & 0<T_{is}<\\ 0 & T_{is}\geq T_{t} \end{array}T_{t}\right.$$

where $u_{s}$ and $u_{t}$ represents SCRI and TCRI, respectively. $T_{is}$ represents the estimated time when obstacles invade the OS’s domain. $T_{t}$ represents the time threshold.

#### 3.1.3. COLREGs

When there is a collision risk between the OS and the obstacle (target ship), the collision situation shall be identified based on the encounter situation identification model [31], and the responsibility for avoidance shall be divided according to the provisions of Articles 13, 14, and 15 of the COLREGs as follows.

(1) Head-on situation. Both the OS and the target ship are given-way ships and should adjust course to starboard to prevent a collision.
(2) Crossing situation. If a starboard crossing situation is formed, the OS is the given-way ship and should adjust course to starboard to prevent a collision. If a port crossing situation is formed, the OS is the stand-on ship. But when the CRI is greater than a certain value and the target ship has not taken collision avoidance maneuvers, the OS can adjust course to the direction with a smaller amplitude to prevent a collision.
(3) Overtaking situation. If the OS overtakes the target ship, the OS is the given-way ship and should adjust course to the direction with a smaller amplitude to prevent a collision. If the target ship overtakes the OS, the OS is the stand-on ship. But when the CRI is greater than a certain value and the target ship has not taken collision avoidance maneuvers, the OS can adjust course to the direction with a smaller amplitude to prevent a collision.

### 3.2. Traditional Artificial Potential Field Algorithm

The APF algorithm, proposed by Khatib [8], is based on the concept of constructing a gravitational potential field around the target point and a repulsive potential field around the obstacle. The OS is simultaneously subjected to both gravitational force and repulsive force to navigate toward the target point.

#### 3.2.1. Gravitational Force

The gravitational force attracts the OS toward the target point, and its magnitude is proportional to the distance between the OS and the target point. The corresponding gravitational potential field function $U_{att}$ is expressed as:

(6) $$U_{att}\;=\;\frac{1}{2}k_{att1}d_{oe}^{2}$$

where $k_{att1}$ is the gravitational coefficient. $d_{oe}$ is a vector that represents the Euclidean distance between the OS and the target point, with the direction is the OS pointing towards the target point.

The gravitational force ($F_{att}$) acting on the OS is obtained from the negative gradient of the gravitational potential field:

(7) $$F_{att}=-\nabla U_{att}=k_{att1}d_{oe}$$

Physically, $F_{att}$ represents the target-directed driving force that guides the OS toward the target point. Mathematically, it constitutes one component of the resultant force in the improved APF framework. Together with the route gravitational force and the repulsive forces generated by target ships and static obstacles, $F_{att}$ is used to determine the resultant force and the desired course of the OS.

In the adopted Cartesian coordinate system, the origin is located at (0,0), the positive *X*-axis points eastward, and the positive *Y*-axis points northward. The components of the gravitational force along the *X*- and *Y*-axes are expressed as:

(8) $$\left\{\begin{array}{c} F_{attx}\;=\;F_{att}\sin\left(\psi_{att}\right)\\ F_{atty}\;=\;F_{att}\cos\left(\psi_{att}\right) \end{array}\right.$$

where $\psi_{att}$ is the angle between gravitational force and the Y-axis direction.

#### 3.2.2. Repulsive Force

The repulsive force, generated by obstacles, pushes the ship away to prevent collisions. Its magnitude increases as the ship approaches an obstacle, ensuring a safe distance is maintained. The repulsive force is inversely related to the distance between the ship and the obstacle, with the corresponding potential field function given in Equation (9).

(9) $$U_{req}=\left\{\begin{array}{cc} \frac{1}{2}k_{req1}{\left(\frac{1}{d_{ob}}-\frac{1}{\rho_{0}}\right)}^{2} & 0\leq d_{ob}\leq \rho_{0}\\ 0 & d_{ob}\geq \rho_{0} \end{array}\right.$$

where $k_{req1}$ is the repulsive coefficient. $d_{ob}$ is the Euclidean distance between the ship and the obstacle, with the direction is the obstacle pointing towards the ship. $\rho_{0}$ is the maximum distance at which an obstacle can affect the ship.

To solve the problem of unreachable, the commonly used repulsive potential field function [32] is in Equation (10).

(10) $$U_{req}=\left\{\begin{array}{cc} \frac{1}{2}k_{req1}{\left(\frac{1}{d_{ob}}-\frac{1}{\rho_{0}}\right)}^{2}{d_{oe}}^{n} & 0\leq d_{ob}\leq \rho_{0}\\ 0 & d_{ob}\geq \rho_{0} \end{array}\right.$$

where ${d_{oe}}^{n}$ is the distance between the OS and the target point, and (n) is a positive real constant that controls the attenuation rate of the repulsive potential with respect to the target distance. Specifically, (n) determines how strongly the repulsive potential is weighted by the distance between the own ship and the target point. A larger value of (n) leads to a faster decay of the repulsive potential as the own ship approaches the target, thereby alleviating the goal non-reachable problem while maintaining obstacle avoidance capability. In this study, *n* = 2 is adopted, as it provides a suitable balance between navigation safety, convergence toward the target, and trajectory smoothness, and has been widely used in artificial potential field-based path planning methods.

The repulsive force ($F_{req}$) acting on the OS is the negative gradient of the repulsive potential field ($U_{req}$), as in Equation (11). The directions of $F_{req1}$ and $F_{req2}$ are the obstacle pointing toward the OS, and the OS pointing toward the target point, respectively.

(11) $$F_{req}=-\nabla U_{req}=\left\{\begin{array}{cc} F_{req1}+F_{req2} & 0\leq d_{ob}\leq \rho_{0}\\ 0 & d_{ob}\geq \rho_{0} \end{array}\right.$$

(12) $$F_{req1}=k_{req1}\left(\frac{1}{d_{ob}}-\frac{1}{\rho_{0}}\right)\frac{{d_{oc}}^{n}}{{d_{ob}}^{2}}$$

(13) $$F_{req2}=\frac{n}{2}k_{req1}{\left(\frac{1}{d_{ob}}-\frac{1}{\rho_{0}}\right)}^{2}{d_{oc}}^{n-1}$$

The components of repulsive force on the X and Y axes are shown in Equation (14).

(14) $$\left\{\begin{array}{c} F_{reqx}=F_{req1}sin(\psi_{req})\\ F_{reqy}=F_{req1}cos(\psi_{req}) \end{array}\right.$$

where $\psi_{req}$ is the angle between the repulsive force and the *Y*-axis direction.

#### 3.2.3. Combined Force

The combined force acting on the OS is the sum of gravitational force and repulsive force, as shown in Equation (15).

(15) $$\left\{\begin{array}{c} F_{x}=F_{attx}+F_{reqx}\\ F_{y}=F_{atty}+F_{reqy} \end{array}\right.$$

**Figure 3:**
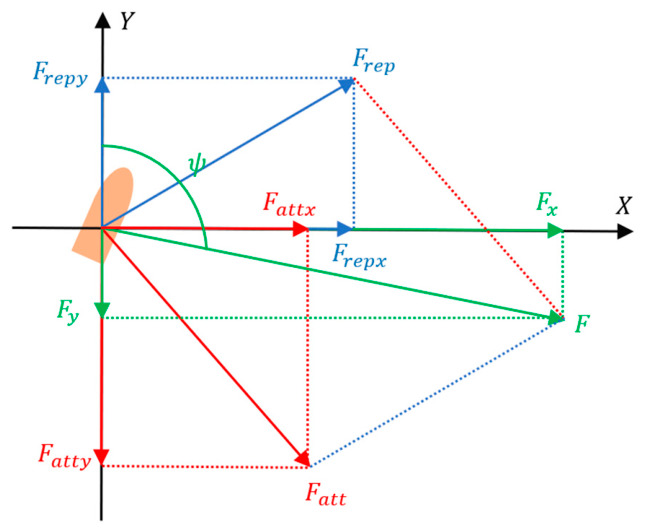
The combined force direction.

The combined force direction (ψ) can be calculated by the components of the combined force on the *X* and *Y* axes, as in Equation (16) and Figure 3.

(16) $$\psi =\left\{\begin{array}{c} arctan\left(\frac{F_{x}}{F_{y}}\right),F_{y}>0\\ \pi +arctan\left(\frac{F_{x}}{F_{y}}\right),F_{x}<0\\ \frac{\pi }{2}\;F_{x}>0,F_{y}=0\\ -\frac{\pi }{2}\;F_{x}<0,F_{y}=0 \end{array}\right.$$

### 3.3. Improved Artificial Potential Field Algorithm

#### 3.3.1. Improvement of Target Point Gravitational Force Function

When the ship is distant from the target point, the target point gravitational force is substantial. At this point, the gravitational force direction and combined force direction are almost aligned, resulting in a combined force direction that is ineffective in avoiding the obstacle. As the ship approaches the obstacle, the repulsive force increases rapidly, but by then, it may be too late to avoid a collision.

**Figure 4:**
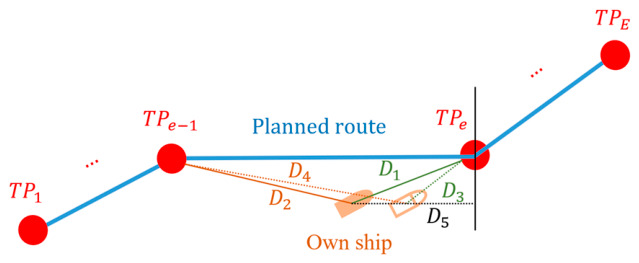
Current target point judgment.

In the study, a distance threshold ($d_{1}$) is introduced to improve the gravitational force function ($U_{att}$) which can allow the ship to take collision avoidance maneuvers earlier. When the distance between the ship and the obstacle exceeds the threshold, the gravitational force becomes constant and no longer increases with distance, as shown in Equation (17).

(17) $$U_{att}=\left\{\begin{array}{c} k_{att1}d_{1},\;\;d_{oe}\geq d_{1}\\ \frac{1}{2}k_{att1}d_{oe},d_{oe}<d_{1} \end{array}\right.$$

where $d_{oe}$ represents the distance between the ship and the target point.

(18) $$d_{oe}=\sqrt{{\left(x_{os}-x_{1}\right)}^{2}+{\left(y_{os}-y_{1}\right)}^{2}}$$

where $\left(x_{os},y_{os}\right)$ represents the coordinates of the ship; $\left(x_{1},y_{1}\right)$ represents the coordinates of the current target point. The method for determining the current target point is as follows:

(1) As in Figure 4, there are a total of E turning points on the planned route. The distances between the ship and all turning points are calculated, with the closest turning point to the ship denoted as ${TP}_{e}$.
(2) The current distances from the ship to ${TP}_{e}\left(D_{1}\right)$ and ${TP}_{e-1}$ $\left(D_{2}\right)$ are calculated, respectively.
(3) The next moment distances from the ship to ${TP}_{e}\left(D_{3}\right)$ and ${TP}_{e-1}$ $\left(D_{4}\right)$ are calculated, respectively, using the line connecting ${TP}_{e-1}$ and ${TP}_{e}$ as the course.
(4) When ($D_{1}-D_{2})(D_{3}-D_{4})>0$, ${TP}_{e+1}$ is the current target point.
(5) The distance from the ship to the turning line $\left(D_{5}\right)$ is calculated.
(6) When ($D_{1}-D_{2})(D_{3}-D_{4})<0$ and $D_{5}\leq 0.3\;mile$, ${TP}_{e+1}$ is the current target point. When ($D_{1}-D_{2})(D_{3}-D_{4})<0$ and $D_{5}>0.3\;mile$, ${TP}_{e}$ is the current target point.

#### 3.3.2. Increase Route Gravitational Force Function

**Figure 5:**
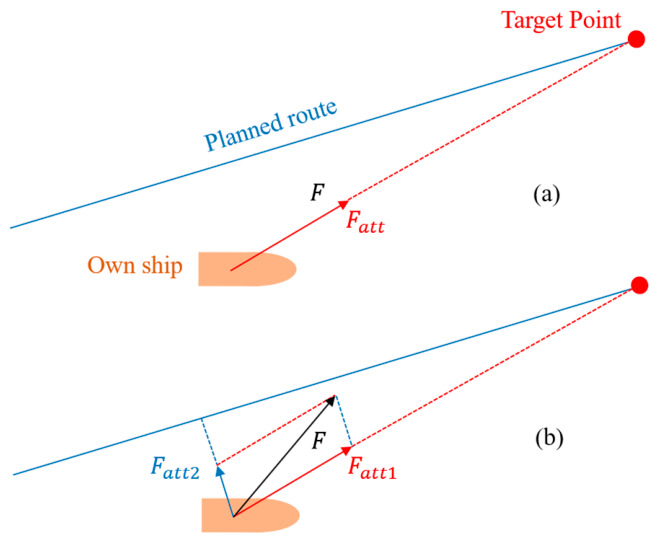
Comparison of navigation trajectories under different potential field functions: (**a**) Traditional APF algorithm; (**b**) Improved APF algorithm with planned route gravitational force.

In the traditional APF algorithm, the ship will navigate directly toward the target point when not influenced by the obstacle repulsive force, which causes the ship to return to the planned route slowly, as in Figure 5a. To address this issue of route deviation, the planned route gravitational force is introduced. When the ship is not influenced by the obstacle repulsive force and the distance from the route exceeds a certain threshold, the ship is influenced not only by the target point gravitational force but also by the route gravitational force. This helps the ship return to the planned route more quickly, as in Figure 5b.

The route gravitational force function is in Equation (19).

(19) $$F_{att2}=\left\{\begin{array}{c} k_{att2}\left(d_{op}+d_{oe}\right),\;d_{op}>d_{2}\\ 0,\;d_{op}<d_{2} \end{array}\right.$$

where $k_{att2}$ is the route gravitational force coefficient. $d_{2}$ is the distance threshold. $d_{op}$ is the distance from the ship to the route, with the ship pointing vertically towards the route, as in Equation (20).

(20) $$d_{op}=\frac{\left|\left(y_{2}-y_{1}\right)x_{os}-\left(x_{2}-x_{1}\right)y_{os}+x_{2}y_{1}-x_{1}y_{2}\right|}{\sqrt{{\left(y_{2}-y_{1}\right)}^{2}+{\left(x_{2}-x_{1}\right)}^{2}}}$$

where $\left(x_{os},y_{os}\right)$ and $\left(x_{2},y_{2}\right)$ represent the coordinates of the OS and the previous target point, respectively. $\left(x_{1},y_{1}\right)$ represents the coordinates of the current target point, which can be judged as the same as in Section 3.3.1.

#### 3.3.3. Improvement of Obstacle Repulsive Force Function

**Figure 6:**
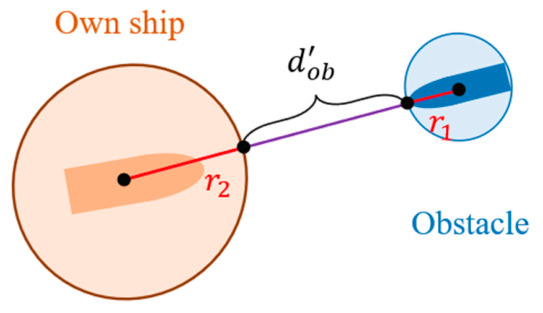
Obstacle repulsive force (ship model).

(1) The traditional repulsive function calculates the distance ($d_{ob}$) between the obstacle and ship based on particle points, instead of considering the shape of the ship and the obstacle, which may result in the ship passing through the obstacle. The ship domain and digital model of obstacles [33] are introduced to solve this problem, as in Figure 6.

The distance after improvement ($d_{ob}^{\prime }$) is in Equation (21).

(21) $$d_{ob}^{\prime }=d_{ob}-r_{1}-r_{2}$$

**Figure 7:**
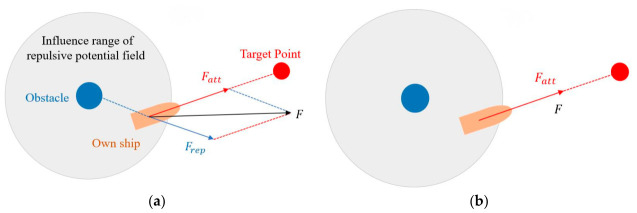
Comparison of obstacle repulsive potential fields: (**a**) Conventional repulsive potential function; (**b**) Improved repulsive potential function integrated with the CRI collision risk model.

(2) In the traditional repulsive function, the ship is influenced by the repulsive force whenever it enters the obstacle’s repulsive potential field, as shown in Figure 7a. However, in maritime practice, it is clear that no collision risk exists between the ship and the obstacle in such cases, and the obstacle does not affect the ship’s movement. To enhance the repulsive potential field function, a CRI model is introduced in this study. This model ensures that the ship is not influenced by the obstacle repulsive force when no collision risk is present, as shown in Figure 7b.

(3) The traditional APF algorithm employs a repulsive function to avoid collisions between ships and obstacles. However, as the ship approaches the obstacle, the repulsive force increases sharply, which may cause disproportionately large directional adjustments and lead to an unstable navigational trajectory. To address this issue, a smoother growth method is adopted to better control the path and directional adjustments.

In summary, the improved obstacle repulsive potential field function is in Equation (22), and the repulsive force is shown in Equation (23).

(22) $$U_{req}=\left\{\begin{array}{c} \frac{1}{2}k_{req1}{\left(\frac{1}{\sqrt{d_{ob}^{\prime }}}-\frac{1}{\sqrt{\rho_{0}}}\right)}^{2}{d_{oe}}^{n}\;u>0\\ 0\;u\leq 0 \end{array}\right.$$

(23) $$F_{req}=-\nabla U_{req}=\left\{\begin{array}{c} F_{req1}+F_{req2}\;u>0\\ 0\;u\leq 0 \end{array}\right.$$

(24) $$F_{req1}=k_{req1}\left(\frac{1}{\sqrt{d_{ob}^{\prime }}}-\frac{1}{\rho_{0}}\right)\frac{{d_{oe}}^{n}}{{d_{ob}^{\prime }}^{1.5}},u>0$$

(25) $$F_{req2}=\frac{n}{2}k_{req1}{\left(\frac{1}{\sqrt{d_{ob}^{\prime }}}-\frac{1}{\rho_{0}}\right)}^{2}{d_{oe}}^{n-1}\;u>0$$

When calculating the repulsive force of obstacles, $d_{oe}$ is assigned a constant value of $d_{1}$. Consequently, the magnitude of the obstacle’s repulsive force depends solely on $d_{ob}^{\prime }$.

#### 3.3.4. Increase Risk Index Repulsive Force Function

**Figure 8:**
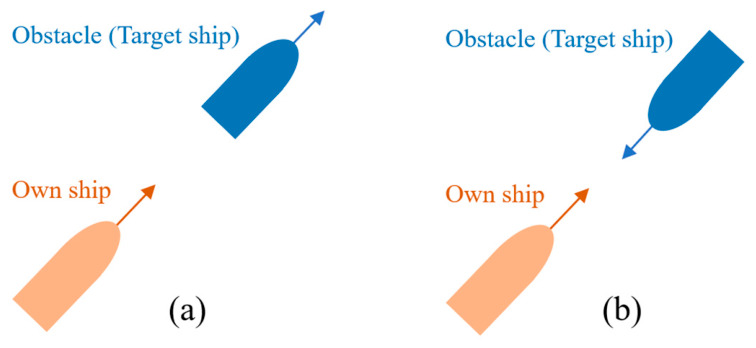
Repulsive force comparison under different encounter scenarios of moving obstacles: (**a**) Overtaking encounter of OS and TS in traditional APF; (**b**) Head-on encounter of OS and TS in traditional APF.

The traditional APF algorithm is primarily designed for static obstacle avoidance and does not account for the motion of obstacles. In Figure 8a and Figure 8b, the own ship (OS) and the target ship (TS) are in an overtaking situation and a head-on situation, respectively. Since the distance between the OS and the TS is the same in both scenarios, the repulsive force experienced by the OS is also the same. However, this does not align with practical navigation, as the situation in Figure 8b is more dangerous. Therefore, the OS should experience a greater repulsive force to accurately reflect the increased level of danger.

Therefore, when considering dynamic obstacle avoidance, it is essential to account for the relative motion between the OS and TS. Relative motion refers to the movement of one object with respect to another, considering both their speeds and directions. The collision risk repulsive force is introduced to describe the relative motion between the OS and the target ship, as in Equation (26).

(26) $$F_{req3}=\left\{\begin{array}{c} k_{req2}\cdot u^{k_{req3}}\cdot d_{oe},d_{oe}<d_{1}\\ k_{req2}\cdot u^{k_{req3}}\cdot d_{1},d_{oe}\geq d_{1} \end{array}\right.$$

where u represents the CRI. $k_{req2}$ and $k_{req3}$ represent the risk index repulsive coefficient. $d_{oe}$ represent the distance between the target point and OS.

#### 3.3.5. Improvement of Repulsive Force Direction

When there is a collision risk between the OS and the obstacle, and the obstacle is dynamic, the requirements of collision avoidance regulations must be considered. The repulsive force directions for static and dynamic obstacles are as follows.

(1) Dynamic obstacles

**Figure 9:**
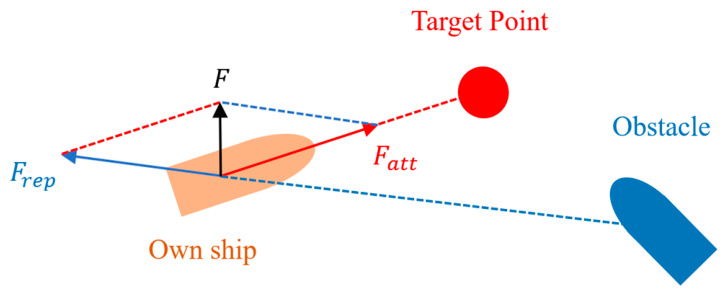
Combined force direction violating COLREGs.

The requirements of the COLREGs are not considered in traditional repulsive force functions. As in Figure 9, when the OS and TS form a starboard crossing situation, the OS should adjust course to starboard to prevent a collision according to COLREGs. However, the combined force direction points to the port, which violates the COLREGs.

In the study, the repulsive force direction is improved considering the COLREGs.

**Figure 10:**
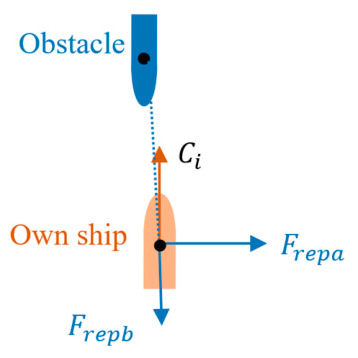
The repulsive force direction (Head-on situation).

Head-on situation. The repulsive force direction points to the right of the OS’s initial course ($C_{i}$), which is the course at the time the collision risk is identified. During the avoidance maneuver, the initial course remains unchanged. The repulsive force direction $F_{repb}$ and $F_{repa}$ before and after improvement is in Figure 10.

**Figure 11:**
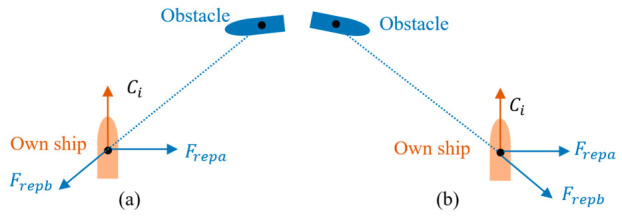
Comparison of repulsive force directions before and after optimization under crossing encounter scenarios: (**a**) Starboard crossing situation; (**b**) Port crossing situation.

Starboard crossing situation. The repulsive force direction points to the right of $C_{i}$. The repulsive force direction before and after improvement is shown in Figure 11a.

Port crossing situation. The OS maintains the current course if the CRI is less than 0.2. The repulsive force direction points to the right of $C_{i}$ if the CRI is greater than 0.2. The repulsive force direction before and after improvement is shown in Figure 11b.

**Figure 12:**
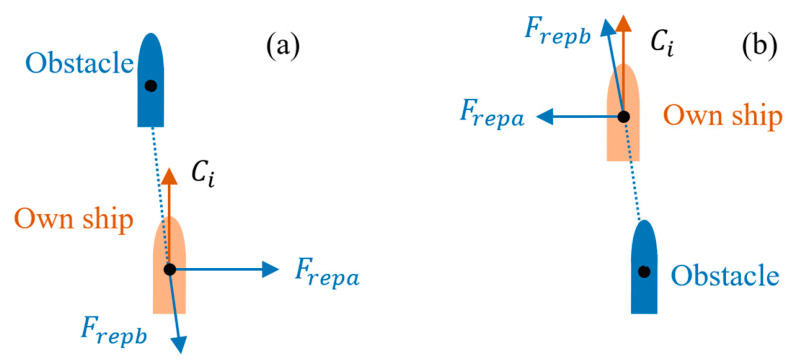
Comparison of repulsive force directions before and after optimization under overtaking encounter scenarios: (**a**) Overtaking situation; (**b**) Being overtaken situation.

Overtaking situation. A straight line with the center of the OS as the origin and $C_{i}$ as the direction is drawn. If the TS is located on the left side of the line, the repulsive force direction points to the right $C_{i}$. Otherwise, the repulsive force direction points to the left of $C_{i}$. The repulsive force direction before and after improvement is shown in Figure 12a.

Being overtaking situation. If the CRI is less than 0.2, the OS maintains its current course. If the CRI is greater than 0.2, a straight line with the center of the OS as the origin and $C_{i}$ as the direction is drawn. If the TS is located on the left side of the line, the repulsive force direction points to the right of $C_{i}$. Otherwise, the repulsive force direction points to the left of $C_{i}$. The repulsive force direction before and after improvement is shown in Figure 12b.

(2) Static obstacles

First, whether the OS has deviated from the planned route is determined. The OS is considered not deviated from the planned route if both of the following conditions are met: The OS is within 100 m of the planned route; The OS’s course deviates by less than 5° from the direction of the planned route.

**Figure 13:**
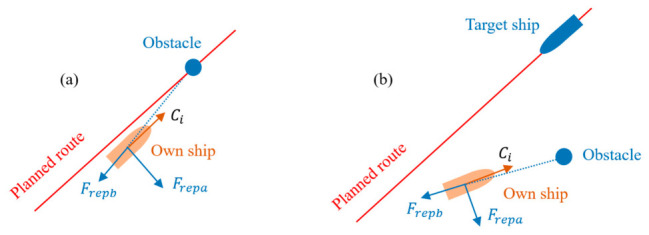
Comparison of repulsive force directions for static obstacles under two navigation states of the own ship: (**a**) Own ship without route deviation; (**b**) Own ship deviating from the planned route.

When the OS has not deviated from the planned route and poses a collision risk with the static obstacle, a straight line is drawn with the center of the OS as the origin and $C_{i}$ as the direction. If the obstacle is located on the left side of the line, the repulsive force direction points to the right of $C_{i}$. Otherwise, the repulsive force direction points to the left of $C_{i}$. The repulsive force direction before and after improvement is shown in Figure 13a.

When the OS deviates from the planned route and poses a collision risk with a static obstacle, it indicates that the OS is affected by other obstacles and cannot resume navigation. As in Figure 13b, the OS is affected by the target ship and cannot resume navigation, the repulsive force direction is perpendicular to $C_{i}$ and points away from the planned route.

### 3.4. Velocity Obstacle Algorithm

**Figure 14:**
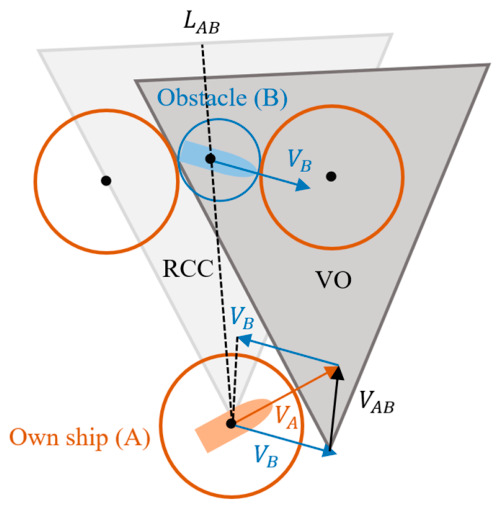
Fundamental principle diagram of the velocity obstacle (VO) algorithm.

The VO algorithm, first proposed by [34] is a widely applied dynamic obstacle avoidance method for unmanned surface vehicles. This algorithm analyses the velocities and positions of both ships and obstacles to predict potential collision zones. The fundamental principle of the algorithm is shown in Figure 14.

A and B represent the ship domain and the obstacle, respectively. $V_{A}$ and $V_{B}$ represent the velocity vectors of the OS and the obstacle, respectively. $V_{AB}$ represents the relative velocity of the OS concerning the obstacle. $L_{AB}$ is a ray that is collinear with the relative velocity $V_{AB}$ at the center of the ship domain. RCC represents the region of potential collision, as in Equation (27).

(27) $$RCC=\left\{V_{AB}|L_{AB}\cap B\neq \emptyset \right\}$$

The unnavigability region is obtained by translating the RCC along the velocity vector of the obstacle, as in Equation (28).

(28) $$VO=RCC⨁V_{B}$$

where ⨁ represents the Minkowski sum operation.

When multiple obstacles are present, the *VO* can be considered as a collection of independent *VO_i_*, as shown in Equation (29).

(29) $$VO={⋃}_{i}^{I}{VO}_{i}$$

In the improved APF algorithm, the OS is only affected by gravitational force after completing collision avoidance maneuvers. However, the direction of gravitational force may again pose a collision risk with the obstacle. After the collision risk is formed, the OS will again take collision avoidance maneuvers. This process will continue to cycle, resulting in path oscillation. The *VO* algorithm is introduced in the study. After the OS completes avoiding the obstacle, the *VO* algorithm is used to judge whether the OS can resume navigation or not. The steps are as follows:

The direction of the combined force, which is the combination of the target point gravitational force and the route gravitational force, is calculated.

The velocity obstacle region based on the velocity obstacle algorithm is calculated.

Whether the combined force direction is within the unnavigability region is determined. If it is not, the OS starts to track the route along the combined force direction. If it is, the OS maintains the combined force direction from the previous moment.

### 3.5. Model of Simulation Ship’s Motion

The research subject is a bulk carrier with a length of 200 m, a width of 27.8 m, and a speed of 17.26 knots. To effectively control the course of the ship, the PID control method which is widely used in marine navigation is employed, as in Equation (30).

(30) $$\delta \left(t\right)=K_{p}\left[e\left(t\right)+\frac{∆t}{T_{i}}\sum_{i=1}^{t}e\left(i\right)+\frac{T_{d}}{∆t}\left(e\left(t\right)-e\left(t-1\right)\right)\right]$$

In this study, an incremental PID control is utilized for adjusting the ship’s course, as in Equation (31).

(31) $$∆\delta \left(t\right)=\delta \left(t\right)-\delta \left(t-1\right)=K_{p}\left(e_{0}-e_{1}\right)+K_{i}e_{0}+K_{d}\left(e_{0}-2e_{1}+e_{2}\right)$$

where $∆\delta \left(t\right)$ represents the change in the rudder angle. $\delta \left(t\right)$ represents the current rudder angle at time t. $\delta \left(t-1\right)$ represents the previous rudder angle at time t−1. $e_{0}$, $e_{1}$ and $e_{2}$ represent the current error signal, previous error signal, and error signal from two time steps ago, respectively. $K_{p}$, $K_{i}$ and $K_{d}$ represent the proportional, integral, and derivative coefficients, respectively.

The response-type nonlinear ship motion model [35] is used to simulate the ship’s movements, as in Equation (32).

(32) $$\left\{\begin{array}{c} \frac{d\dot{\psi }}{dt}=\frac{K}{T}(\delta -\alpha \dot{\psi }+\beta {\dot{\psi }}^{3})\\ \frac{d\psi }{dt}=\dot{\psi }\; \end{array}\right.$$

where K and T represent the turning index and the follow-up index, respectively, reflecting the ship’s motion characteristics in response to rudder angle input. A larger K value indicates better turning ability, while a smaller T value indicates better follow-up performance. In this study, the values of K and T are set to 0.38 and 297.75, respectively. Additionally, the parameters α and β are set to 11.95 and 23,928.91, respectively.

## 4. Simulation Test Results and Analysis

An efficient and scalable experimental platform based on Qt5 was developed in this study to validate the effectiveness of the improved APF algorithm in path planning. The platform was designed to simulate various navigation scenarios by configuring different types of dynamic obstacles, thereby enabling a comprehensive evaluation of the path-planning performance of the proposed algorithm. All simulation experiments were conducted on a computer equipped with an Intel Core i7-13700H processor and 16 GB of RAM (Santa Clara, CA, USA), running the 64-bit Windows 11 operating system. The experimental platform was developed using Qt 5.15.2 and compiled with the MinGW8.1.0 64-bit compiler. Unless otherwise stated, all computational-time results reported in this section were obtained under the above hardware and software configuration.

### 4.1. Parameter Settings

**Figure 15:**
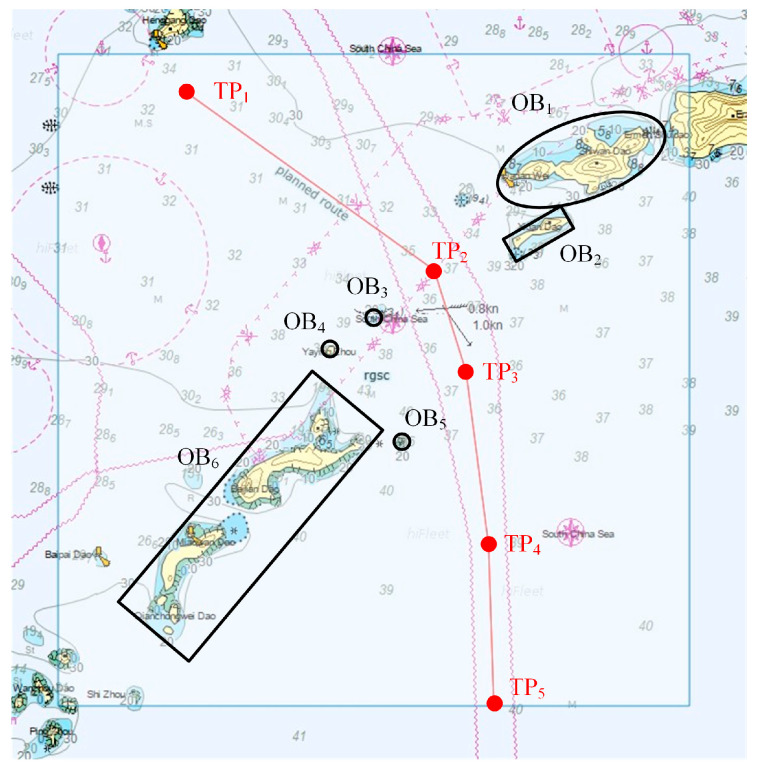
Studied water area.

The studied water area and planned route are shown in Figure 15. The parameter values of the improved APF algorithm are in Table 1.

The parameter values of the improved APF algorithm are in Table 1.

### 4.2. Scenario 1: Head-On Situation

A typical head-on situation is set up in Scenario 1. The parameters of both the OS and TS are in Table 2.

**Figure 16:**
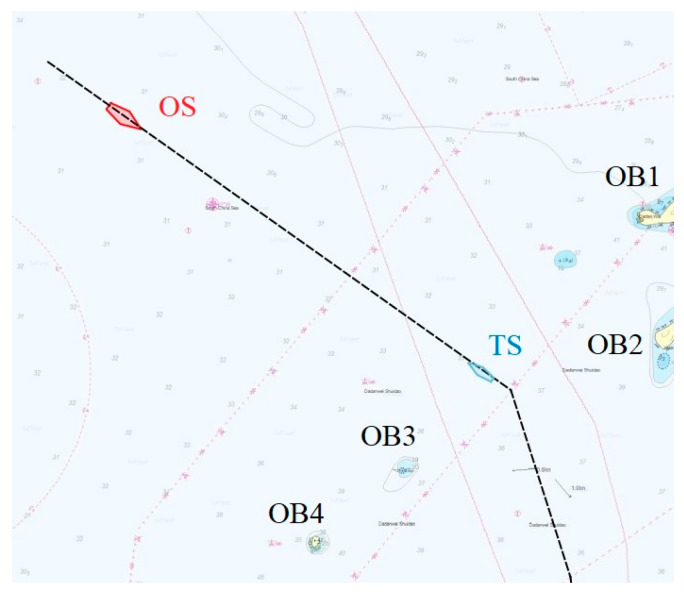
Initial position (Scenario 1).

**Figure 17:**
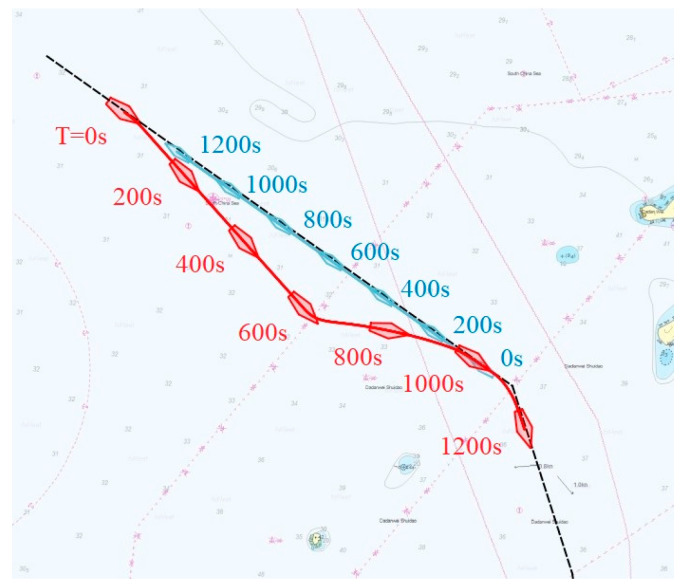
Planned path (Scenario 1).

The initial positions of the OS and TS are in Figure 16, the red and blue represent the OS and TS, respectively. The planned path is in Figure 17.

At 0 s, the OS is on a course of 125.66°, with a distance of 21.14 m from the planned route. The OS is currently unable to resume navigation after judgment. The distance between the OS and TS is 8721.55 m, with a CRI of 0.57. According to the encounter situation identification model, the OS and TS have formed a head-on situation. The OS is a given-way ship and should take collision avoidance maneuvers. The OS’s target course is the combined force direction, which is the superposition of the target point gravitational force, risk index repulsive force, and obstacle repulsive force.

At 65 s, the OS is on a course of 138.52°, with a distance of 89.63 m from the planned route. The OS is currently unable to resume navigation after judgment. The CRI between the OS and TS is 0, while between the OS and OB3 is 0.34. The OB3 is the most dangerous obstacle, and it is a static obstacle. If the OS maintains the current course, an opportunity for resuming navigation is projected to be available after 527 s, while the OB3 will invade the OS’s domain after 849 s. Therefore, the OS maintains the current course and waits for the opportunity to resume navigation.

At 593 s, the OS is on a course of 138.52°, with a distance of 1132.20 m from the planned route. The OS starts to resume navigation after judgment.

**Figure 18:**
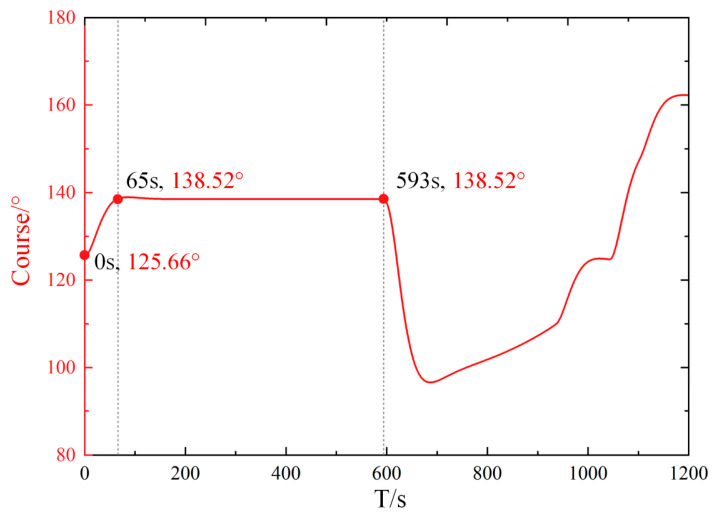
Course (Scenario 1).

**Figure 19:**
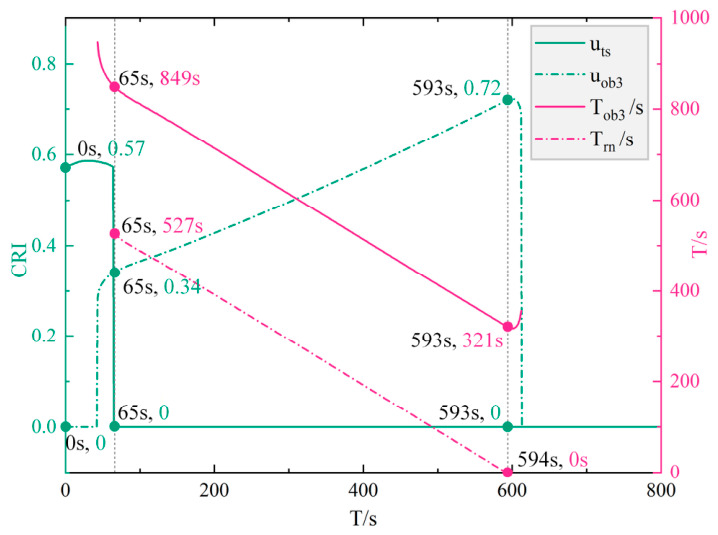
CRI, $T_{rn}$ and $T_{ob3}$ (Scenario 1).

**Figure 20:**
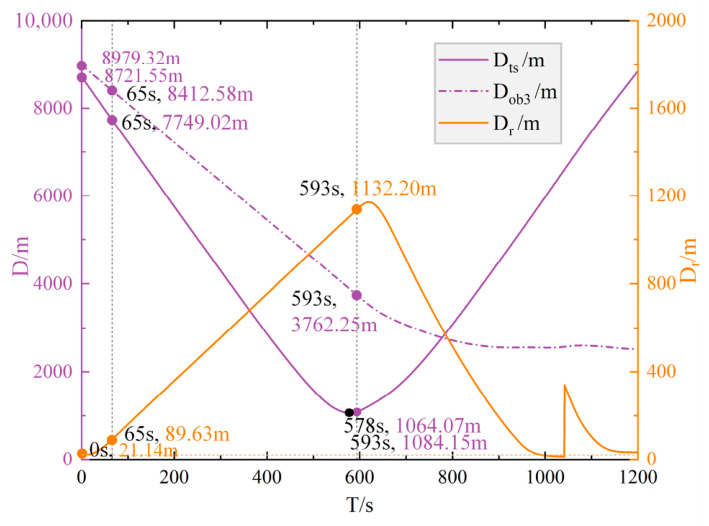
Distance (Scenario 1).

The course of the OS is in Figure 18. The estimated resumption time ($T_{rn}$), the time when OB3 invades the OS’s domain ($T_{ob3}$), the CRI between the OS and the TS ($u_{ts}$), the CRI between the OS and OB3 ($u_{ob3}$) is in Figure 19. The distance between the OS and OB3 ($D_{ob3}$), as well as the distance between the OS and the planned route ($D_{r}$), are in Figure 20.

### 4.3. Scenario 2: Crossing Situation

A typical crossing situation is set up In Scenario 2. The parameters of both the OS and TS are in Table 3.

**Figure 21:**
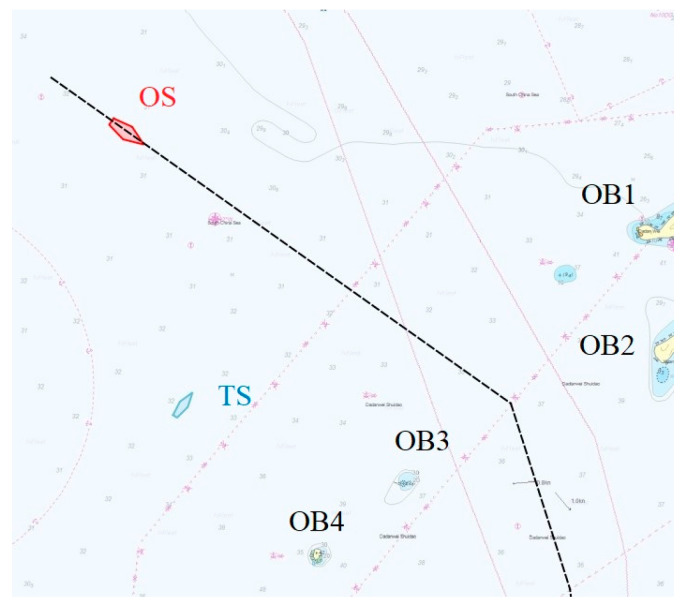
Initial position (Scenario 2).

**Figure 22:**
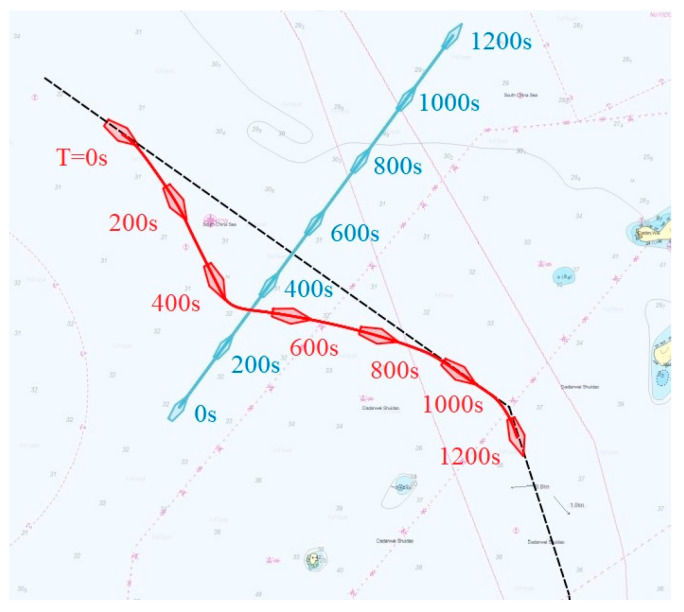
Planned path (Scenario 2).

The initial positions of the OS and TS are in Figure 21, the red and blue represent the OS and TS, respectively. The planned path is in Figure 22.

At 0 s, the OS is on a course of 125.66°, with a distance of 21.14 m from the planned route. The OS is currently unable to resume navigation after judgment. The distance between the OS and the TS is 5621.74 m, with a CRI of 0.66. According to the encounter situation identification model, the OS and TS have formed a crossing situation. The OS is a given-way ship and should take collision avoidance maneuvers. The OS’s target course is the combined force direction, which is the superposition of the target point gravitational force, risk index repulsive force, and obstacle repulsive force.

At 228 s, the OS is on a course of 153.59°, with a distance of 632.19 m from the planned route. The OS is currently unable to resume navigation after judgment. The CRI between the OS and TS is 0, while between the OS and OB4 is 0.40. The OB4 is the most dangerous obstacle, and it is a static obstacle. If the OS maintains its current course, an opportunity for resuming navigation is projected to be available after 190 s, while the OB4 will invade the OS’s domain after 750 s. Therefore, the OS maintains the current course and waits for the opportunity to resume navigation.

At 418 s, the OS is on a course of 153.60°, with a distance of 1431.60 m from the planned route. The OS starts to resume navigation after judgment.

**Figure 23:**
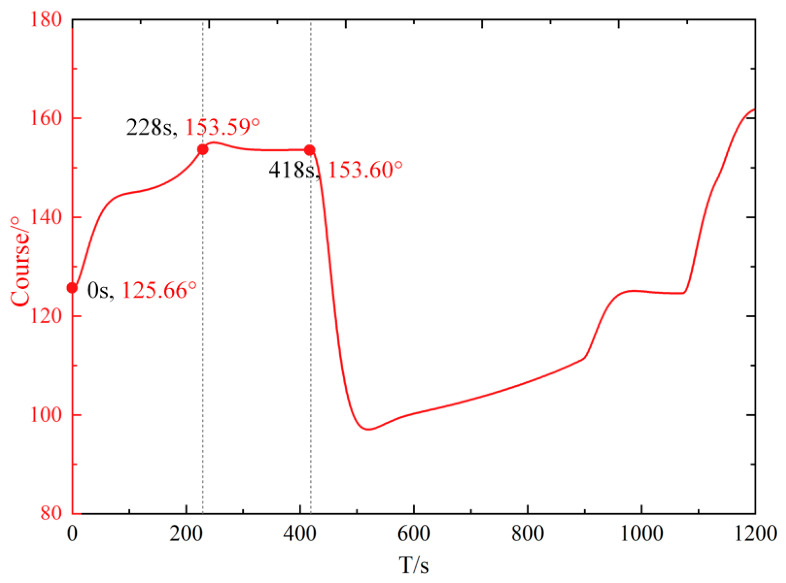
Course (Scenario 2).

**Figure 24:**
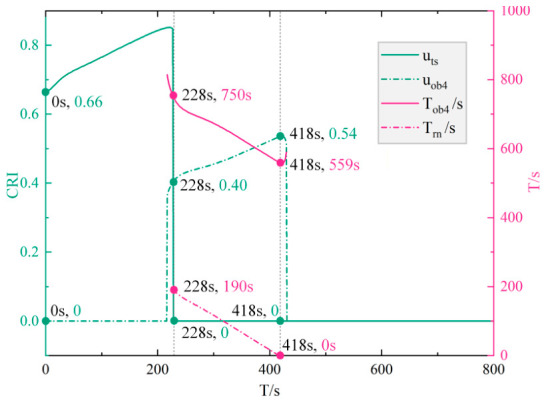
CRI, $T_{rn}$ and $T_{ob4}$ (Scenario 2).

**Figure 25:**
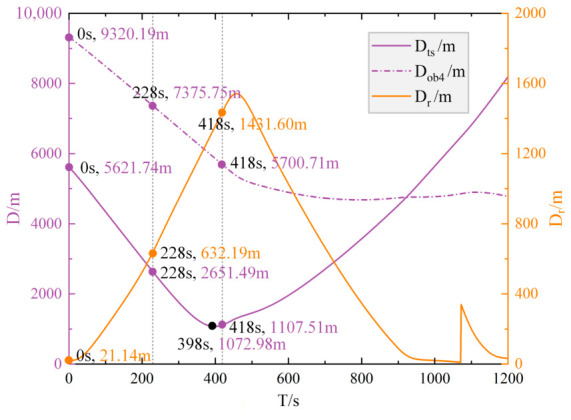
Distance (Scenario 2).

The course of the OS is in Figure 23. The estimated resumption time ($T_{rn}$), the time when OB4 invades the OS’s domain ($T_{ob4}$), the CRI between the OS and the TS ($u_{ts}$), the CRI between the OS and OB4 ($u_{ob4}$) is in Figure 24. The distance between the OS and OB4 ($D_{ob4}$), as well as the distance between the OS and the planned route ($D_{r}$), are in Figure 25.

### 4.4. Scenario 3: Multiple Dynamic Obstacles

A complex multi-ship encounter situation is established in Scenario 3. TS1, TS2, and TS3 maintain constant course and speed, while TS4 maneuvers. The parameters of both the target ships and the OS are detailed in Table 4.

**Figure 26:**
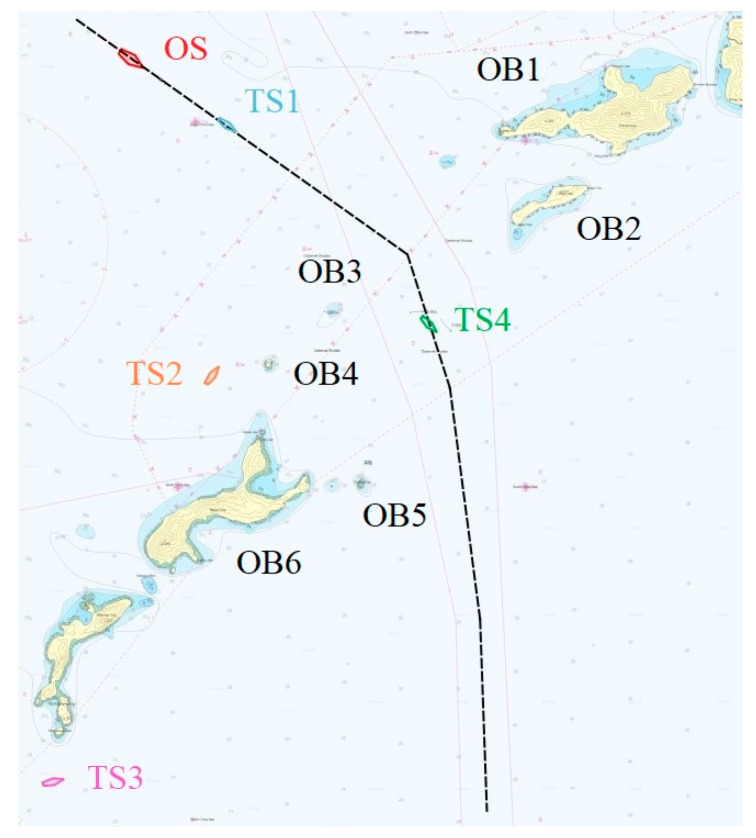
Initial position (Scenario 3).

**Figure 27:**
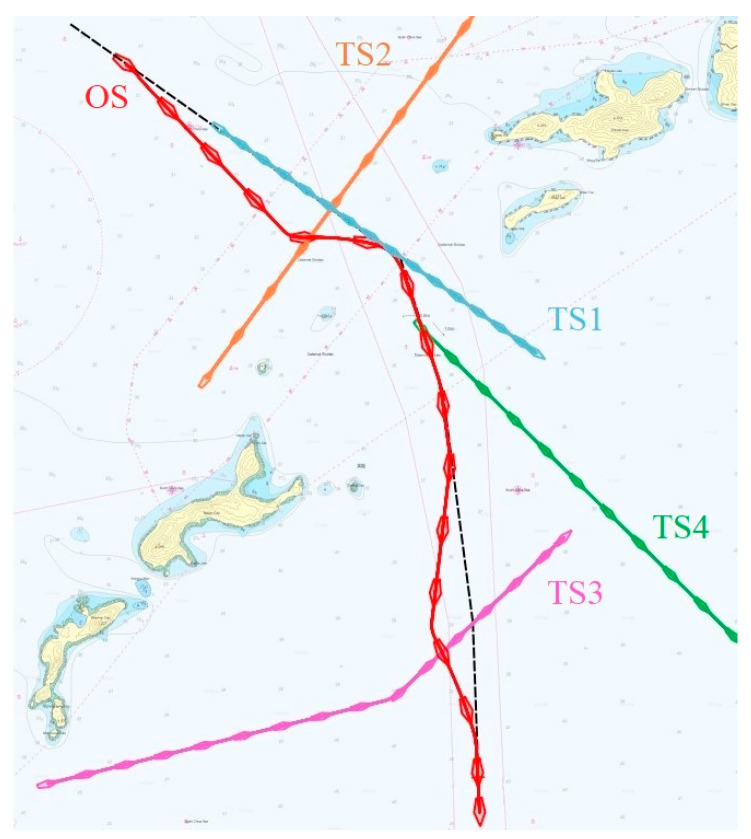
Planned path (Scenario 3).

The initial positions of the OS and TS are shown in Figure 26. The planned path is in Figure 27.

At 0 s, the OS is on a course of 125.66°, with a distance of 21.14 m from the planned route. The OS is currently unable to resume navigation after judgment. The distance between the OS and the TS1 is 3257.93 m, with a CRI of 0.64. The distance between the OS and TS2 is 9171.38 m, with a CRI of 0.44. TS1 is the most dangerous ship, which forms an overtaking situation with the OS. The OS is a given-way ship and should take collision avoidance maneuvers. The OS’s target course is the combined force direction, which is the superposition of the target point gravitational force, risk index repulsive force, and obstacle repulsive force. At 31 s, the OS is on a course of 136.88°, with a distance of 41.89 m from the planned route. The OS is currently unable to resume navigation after judgment. The CRI between the OS and all target ships is 0, while the CRI between the OS and OB3 is 0.31. If the OS maintains its current course, an opportunity for resuming navigation is projected to be available after 683 s, while the OB3 will invade the OS’s domain after 906 s. Therefore, the OS maintains the current course and waits for the opportunity to resume navigation. At 716 s, the OS is on a course of 136.88°, with a distance of 1254.74 m from the planned route. The OS starts to resume navigation after judgment.

At 1710 s, TS3 starts to alter course. At 1731 s, the OS is on a course of 172.28°, with a distance of 33.31 m from the planned route. The OS is currently unable to resume navigation after judgment. The distance between the OS and TS3 is 7357.87 m, with a CRI of 0.51. According to the encounter situation identification model, the OS and TS3 have formed a crossing situation. The OS is a given-way ship and should take collision avoidance maneuvers. The OS’s target course is the combined force direction, which is the superposition of the target point gravitational force, risk index repulsive force, and obstacle repulsive force. At 1810 s, the course of TS3 is stable at 46.3°. At 1967 s, the OS is on a course of 186.61°, with a distance of 439.38 m from the planned route. The OS is currently unable to resume navigation after judgment. The CRI between the OS and all target ships is 0. The OS maintains the current course and waits for the opportunity to resume navigation. At 2282 s, the OS is on a course of 186.61°, with a distance of 1126.71 m from the planned route. The OS starts to resume navigation after judgment. At 2926 s, the OS has arrived at the destination of the planned route.

**Figure 28:**
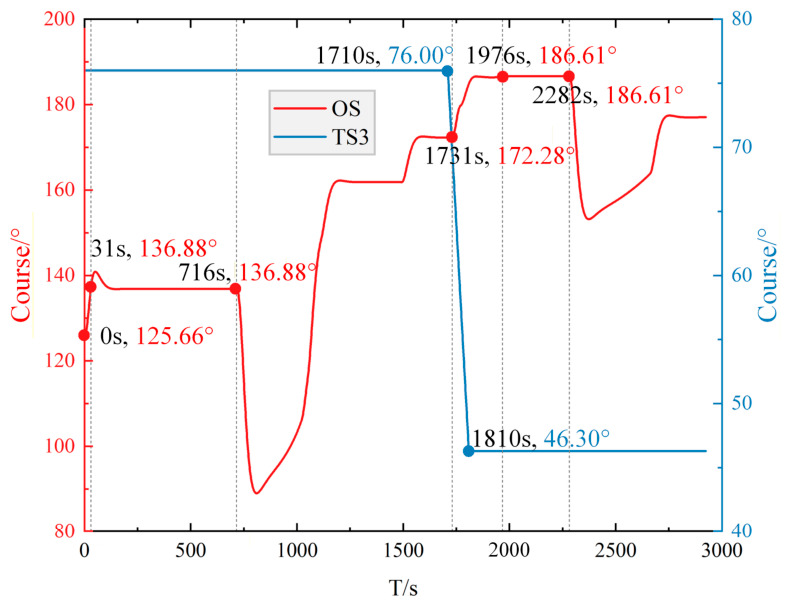
Course (Scenario 3).

**Figure 29:**
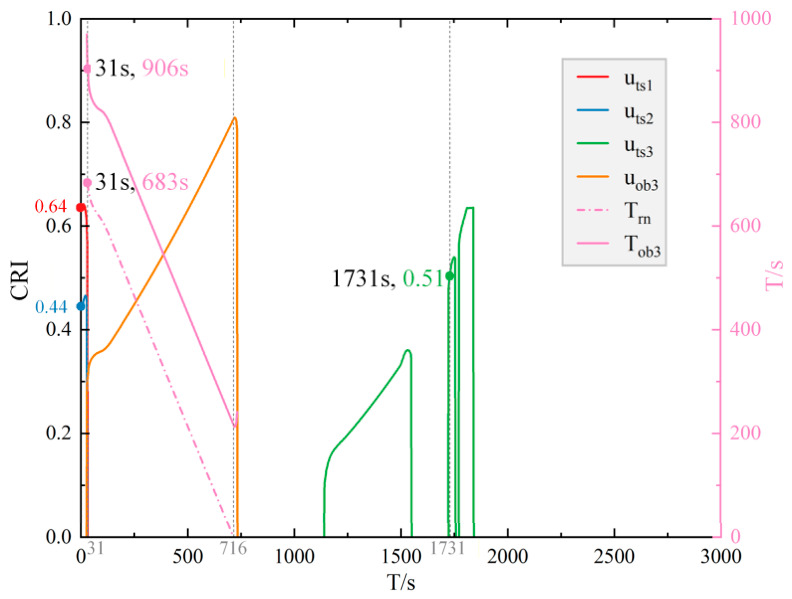
CRI, $T_{rn}$ and u (Scenario 3).

**Figure 30:**
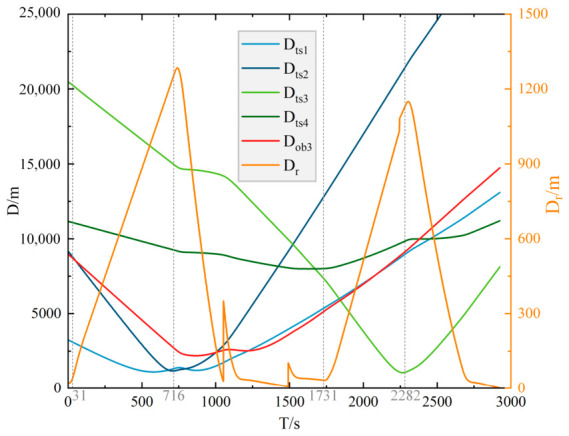
Distance (Scenario 3).

The course of the OS and TS3 is in Figure 28. The estimated resumption time ($T_{rn}$), the time when OB3 invades the OS’s domain ($T_{ob3}$), the CRI between the OS and the TS ($u_{ts1}$, $u_{ts2}$, $u_{ts3}$), the CRI between the OS and OB3 ($u_{ob3}$) is in Figure 29. The distance between the OS and the obstacle ($D_{ts1}$, $D_{ts2}$, $D_{ts3}$, $D_{ob3}$), as well as the distance between the OS and the planned route ($D_{r}$), are in Figure 30.

**Figure 31:**
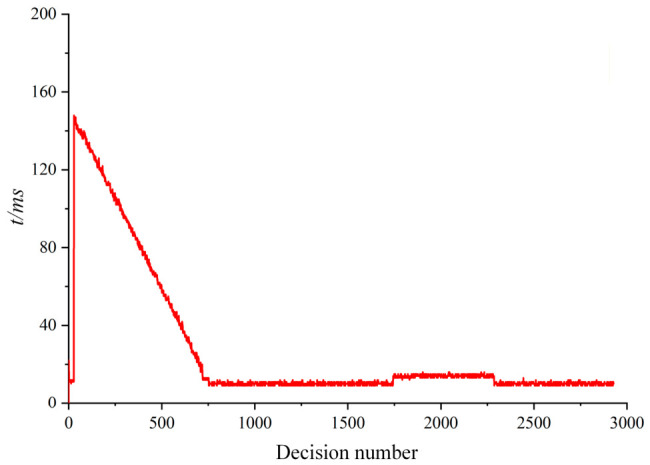
Decision time for each decision-making cycle of the proposed algorithm.

The time required for each decision of the algorithm is shown in Figure 31. From the figure, it can be observed that the maximum decision time is 148 ms (0.148 s), which indicates that the proposed method can achieve real-time collision avoidance decision-making.

Figure 31 shows the computational time required for each decision-making cycle of the proposed algorithm. The horizontal axis represents the decision-cycle number, and the vertical axis represents the computational time of each cycle in milliseconds. The maximum decision time is 148 ms.

### 4.5. Comparative Experiment

In comparative experiment 1, the method proposed in this paper is compared with the artificial potential field method that does not consider COLREGs. The parameters of both the target ships and the OS are detailed in Table 5.

**Figure 32:**
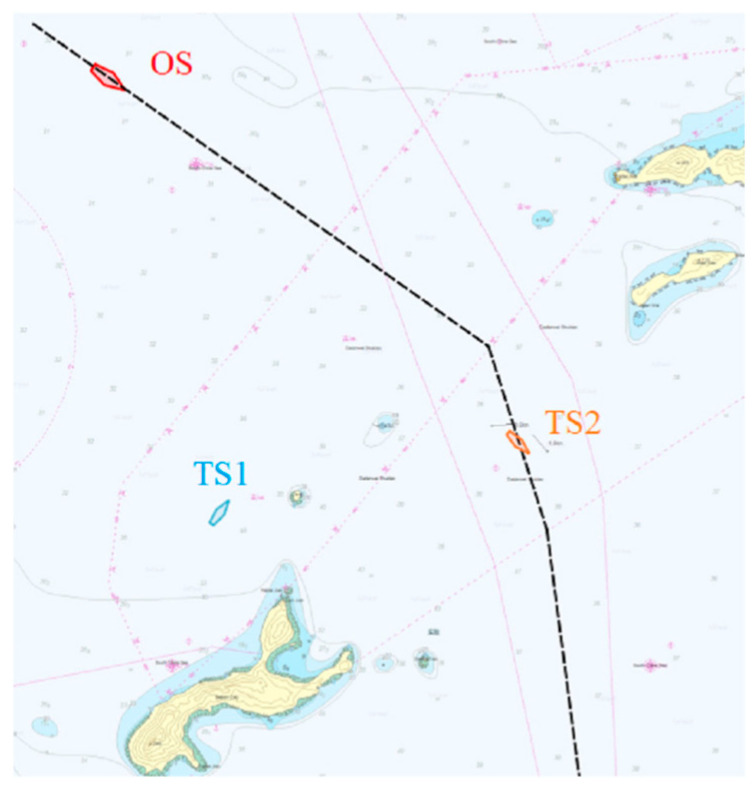
Initial position.

**Figure 33:**
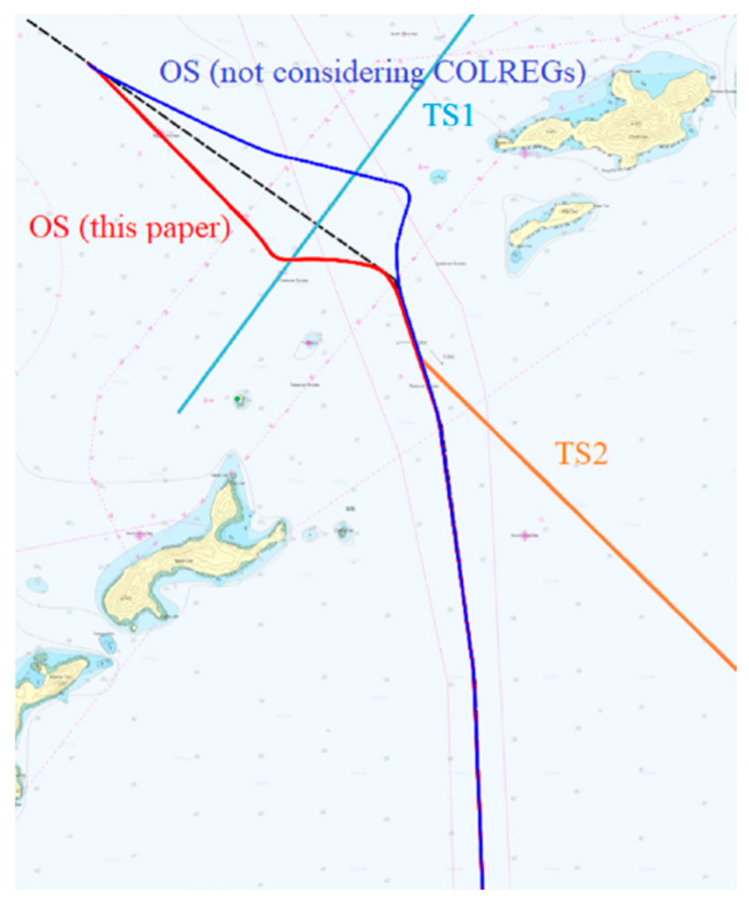
Planned path.

The initial positions of the OS and TS are shown in Figure 32. The paths obtained by different methods are shown in Figure 33.

From the figure, it can be seen that the OS has formed a starboard crossing situation with TS1. According to COLREGs, the OS should alter course to the starboard to avoid the target ship. However, the artificial potential field method that does not consider COLREGs failed to comply with COLREGs in this situation. The method proposed in this article follows COLREGs and can accurately determine and execute reasonable avoidance actions, effectively avoiding collision risks.

### 4.6. Experimental Analysis

In Scenario 1, where the OS and TS are in a head-on situation, the OS adjusts its course 12.86° to starboard to avoid collision. In Scenario 2, where the OS and TS are in a crossing situation, the OS adjusts its course 27.93° to starboard to avoid collision. In Scenario 3, during the first collision avoidance maneuver, TS1 is identified as the most dangerous ship and forms an overtaking situation with the OS. The OS adjusts its course 11.22° to starboard to avoid collision. In the second collision avoidance maneuver of Scenario 3, the OS and TS are in a crossing situation, the OS adjusts its course 14.33° to starboard to avoid a collision. In summary, in Scenarios 1, 2, and 3, the direction of the OS’s course alteration complies with the COLREGs, and the magnitude of the course alteration also meets the standards of maritime practice.

The closest distance between the OS and the target ship in Scenarios 1, 2, and 3 are 1064.07 m, 1072.98 m, and 1109.66 m respectively. The result indicates that the planned paths effectively prevent any intrusion of the target ship into the OS’s domain.

In Scenario 3, the OS simultaneously faces collision risks with both TS1 and TS2 at the initial moment. The OS is capable of identifying the most dangerous target ship (TS1) and calculating the repulsive force based on this ship. When the motion state of TS3 changes, the OS can immediately respond and replan its path accordingly. This demonstrates that the proposed method effectively identifies the most dangerous ship and adapts to the random movements of target ships.

The simulation results show that the variations in the distance between the OS and the target ship, as well as the heading changes in the OS, are smooth, indicating that the proposed method effectively mitigates path oscillations and avoids local minima during the simulated navigation scenarios.

The comparative simulation results indicate that the proposed method outperforms the conventional APF method in the tested scenarios by generating collision avoidance paths that comply with COLREGs while effectively reducing collision risks.

## 5. Conclusions

In this study, an improved artificial potential field (APF)-based path planning method integrated with the velocity obstacle (VO) algorithm was proposed for autonomous ship collision avoidance. Three groups of simulation experiments were conducted to evaluate the performance of the proposed method under different encounter scenarios. The simulation results demonstrate that the proposed method enables the own ship to generate collision avoidance paths that comply with COLREGs while successfully reaching the planned destination. Compared with the conventional APF method, the proposed approach exhibits improved path smoothness, effectively avoids local minima, and provides better collision avoidance performance in the simulated scenarios. In addition, by incorporating the VO algorithm into the APF framework, the proposed method improves the feasibility of the generated collision avoidance decisions while maintaining satisfactory real-time performance.

The present study has several limitations. First, the proposed method has been validated only through simulation experiments, and its effectiveness in real maritime environments has not yet been verified using intelligent ships or full-scale field tests. Therefore, the practical applicability of the proposed method requires further experimental validation. Second, environmental disturbances such as wind, currents, and waves are not considered in the current algorithm. Third, speed adjustment maneuvers are not incorporated into the collision avoidance strategy. Future work will focus on conducting real-world experiments using intelligent ships, incorporating environmental disturbances into the decision-making framework, and extending the proposed method to support coordinated course and speed maneuvering. Moreover, the current evaluation mainly focuses on the effectiveness of the proposed method in representative navigation scenarios. Statistical analyses based on repeated trials, uncertainty quantification, and sensitivity evaluation will be considered in future work to further assess the robustness of the proposed method.

## Figures and Tables

**Table 1:** APF algorithm parameters.

Parameter	Value	Parameter	Value
$k_{att1}$	10	$k_{req1}$	100
$k_{att2}$	2	$k_{req2}$	4
$\rho_{0}$	10,000 m	$k_{req3}$	0.89
$d_{1}$	5000 m	n	2.5
$d_{2}$	100 m		

**Table 2:** Experimental parameters for the head-on encounter situation.

Ship	Lon (°)	Lat (°)	Course (°)	Speed (kn)	Length (m)
OS	114.027442 E	22.002458 N	125.66	17.26	200
TS	114.090957 E	21.959922 N	305.60	12.00	100

**Table 3:** Experimental parameters for the crossing situation.

Ship	Lon (°)	Lat (°)	Course (°)	Speed (kn)	Length (m)
OS	114.027442 E	22.002458 N	125.66	17.26	200
TS	114.037570 E	21.956579 N	36.00	15.00	100

**Table 4:** Experimental parameters for the Multiple Dynamic Obstacles.

Ship	Lon (°)	Lat (°)	Course (°)	Speed (kn)	Length (m)
OS	114.027442 E	22.002458 N	125.66	17.26	200
TS1	114.051475 E	21.986972 N	125.60	7.20	100
TS2	114.047698 E	21.928396 N	36.00	15.00	100
TS3	114.007873 E	21.832818 N	76.00	11.00	100
TS4	114.101943 E	21.940179 N	135.00	12.00	100

**Table 5:** Experimental parameters (Comparison with APF without considering COLREGs).

Ship	Lon (°)	Lat (°)	Course (°)	Speed (kn)	Length (m)
OS	114.027442 E	22.002458 N	125.66	17.26	200
TS1	114.051475 E	21.986972 N	125.60	7.20	100
TS2	114.047698 E	21.928396 N	36.00	15.00	100

## Data Availability

The original contributions presented in this study are included in the article. Further inquiries can be directed to the corresponding authors.
